# Solubility and Bioavailability Enhancement of Poorly Aqueous Soluble Atorvastatin: *In Vitro*, *Ex Vivo*, and *In Vivo* Studies

**DOI:** 10.1155/2014/463895

**Published:** 2014-06-03

**Authors:** Madhuri S. Rodde, Ganesh T. Divase, Tejas B. Devkar, Avinash R. Tekade

**Affiliations:** Department of Pharmaceutics, Rajarshi Shahu College of Pharmacy & Research, Tathawade, Pune 411033, India

## Abstract

The objective of this investigation was to improve the solubility of the poorly water soluble drug atorvastatin (ATR), using solid dispersion (SD) techniques, with Neem Gum (NG) as a hydrophilic carrier. The effects of the polymer concentration and method of preparation on the solubility and dissolution rate were studied. The results showed that the solubility of ATR increases with increasing NG concentration. However, dissolution rate of ATR from its SD was dependent on the method used to prepare SD. An *in vitro* drug release study revealed that the solvent evaporation technique is a more convenient and effective method of preparing SD than kneading method. The SD was characterized using DSC, SEM, and XRD study. An *in vivo* study was performed in which the 3-hydroxy-3-methyl-glutaryl-coenzyme A (HMG CoA) reductase inhibition activity was measured. A significant reduction in HMG CoA reductase activity was observed with SD of ATR compared with the plain drug. An *ex vivo* absorption study was carried out using modified apparatus developed in our laboratory. The *in vitro* drug release and *in vivo* and *ex vivo* studies clearly demonstrated the potential of hydrophilic NG in enhancing the solubility, dissolution rate, and bioavailability of ATR.

## 1. Introduction

Drugs that are poorly soluble in water are associated with slower drug absorption, eventually leading to inadequate and variable bioequivalence [1]. Drugs are classified on the basis of their solubility and permeability characteristics. The Biopharmaceutical Classification System (BCS) categorizes drugs in four classes: Class I, Class II, Class III, and Class IV. Drugs belonging to BCS Class II are poorly soluble in water, with high permeability [2, 3], and thus are ideal candidates for enhancing bioavailability by simply enhancing solubility. Atorvastatin (ATR) is a BCS Class II drug. Used as a hypolipidemic synthetic agent, it is an inhibitor of 3-hydroxy-3-methylglutaryl-coenzyme A reductase (HMG CoA), which catalyses the conversion of HMG CoA to mevalonate, an early rate limiting step in the cholesterol biosynthesis pathway [4]. Chemically it is (3R, 5R)-7-[2-(4-fluorophenyl)-3-phenyl-4-(phenylcarbamoyl)-5-(propan-2-yl)-1-H-pyrrol-1-yl]-3,5-dihydroxyheptanoic acid. It is insoluble in aqueous solutions with pH values less than 4; very slightly soluble in distilled water, phosphate buffer of pH 7.4, and acetonitrile; slightly soluble in ethanol; and freely soluble in methanol [5, 6]. ATR has a plasma half-life of 18–24 hours, much higher than that of other statins. Poor oral bioavailability of ATR (12%) is mainly attributed to its low aqueous solubility (0.1 mg/mL) and its crystalline nature [7].

Enhancement of the dissolution rate of drugs that are poorly soluble in water to improve their oral bioavailability is a challenging task [8]. One successful approach in this direction is the use of solid dispersions (SD). The term “solid dispersions” has been used to describe a family of dosage forms in which molecules of a drug are dispersed in a biologically inert carrier, usually with the aim of enhancing oral bioavailability. It has been reported that dissolution rates may be improved when molecules of drugs are dispersed in polymeric carriers [9]. Fusion and solvent evaporation are the methods most widely used to prepare SD to convert a substance in a crystalline form to an amorphous state either partially or completely so that the solubility and bioavailability are increased [1, 10]. However, some problems are frequently encountered in producing SD, such as difficulties in scaling up, physical instability of the dispersions, and estimating the amount of carrier needed to facilitate the desired increase in the drug release rate. Micronization is commonly used to increase the solubility and dissolution rate of drugs. But micronized drugs tend to agglomerate as a result of their hydrophobicity, thus reducing their available surface area [11]. Agglomeration can be avoided by combining drugs with hydrophilic carriers [12, 13], which may result in a total or partial loss of crystallinity, thereby increasing the solubility and dissolution rate significantly [14].

A number of techniques have been developed to address the challenges associated with low aqueous solubility. These include thin film freezing [15], lyophilization [16], evaporative precipitation into aqueous solutions [17], spray drying [18], cospray drying [19], melt mixing [20], dry coating with magnesium and sodium stearate [21], and surface solid dispersion [22]. Although vast numbers of hydrophilic carriers have been described, alternative carriers are constantly being sought for use in industrial applications and for reducing production costs and toxic effects. Therefore, many natural polymers have been evaluated. The use of a natural polymer with low viscosity values and a high swelling index offers advantages over synthetic polymers [23]. Natural polymers are more beneficial because of their biocompatibility and easy availability [24]. Neem Gum (NG) is a natural polymer that is obtained from neem* Azadirachta indica* trees. The neem is also known as the Indian lilac and belongs to the family Meliaceae [25]. NG has been investigated for use as a tablet binder [26] and mucoadhesive agent [27]. NG is widely used because of its high swelling index, high water retention capacity, digestible nature, binding ability, and easy availability.

To the best of our knowledge, this is the first study in which the use of NG for preparing SD to enhance the solubility and bioavailability of the poorly soluble drug ATR has been explored. The influence of the concentration of NG and the method of preparation of SD on the solubility and dissolution rate was studied. The apparent solubility was investigated, and* in vitro* dissolution studies were performed. SEM, DSC, XRD,* in vivo*, and* ex vivo* studies were also carried out. Modified apparatus developed in our laboratory was used to carry out* ex vivo* absorption studies using everted chicken ileum to predict precisely the phenomena of dissolution and absorption from SD formulations [28].

## 2. Materials and Methods

ATR was obtained as a gift sample from Zydus Cadila Healthcare, Ahmedabad, India. NG was collected from neem trees from the Indapur region of Maharashtra, India. All other materials used were of analytical grade.

### 2.1. Purification of NG

The NG was purified using a method that has been described previously. The gum was dried well and powdered using mortar and pestle. The powdered gum was passed through a 100# sieve after which it was solubilized in distilled water. The concentrated solution of the gum was precipitated using ethanol. The precipitate was separated and dried at 60°C. The resultant dried gum was powdered again and passed through a 100# sieve, and it was then stored in an airtight container for use [26].

### 2.2. Characterization of NG

#### 2.2.1. Swelling Index

NG (1 g) was weighed accurately and transferred to a 100 mL measuring cylinder. The initial volume of the powder was noted. Then the gum was dispersed thoroughly in distilled water by vigorous shaking. The measuring cylinder was maintained for 24 hours at ambient temperature and humidity. The volume occupied by the NG sediment after 24 hours was noted. The swelling index, expressed as a percentage, was calculated according to the following equation:

(1) $$\begin{array}{c} \text{SI}=\left[\left(\frac{{X_{1}-X}_{0}}{X_{0}}\right)\right]\times 100, \end{array}$$

where *X*_0_ is the initial height of the powder in the graduated cylinder and *X*_1_ is the height of the swollen gum after 24 hours.

#### 2.2.2. Water Retention Capacity

After the swelling index study was carried out, the contents of the measuring cylinder were filtered using a muslin cloth, and the water was allowed to drain completely into a dry 100 mL graduated cylinder. The volume of the water collected was noted. The water retained by the sample was determined as the difference between the original volume of the mucilage and the volume of the drained water. The amount of water retained per unit volume of a polysaccharide is referred to as its water retention capacity or water absorption capacity [29].

#### 2.2.3. Viscosity Measurement

The viscosity of a 1% aqueous NG solution was measured according to USP specifications using a Brookfield DV-E viscometer.

#### 2.2.4. Angle of Repose

The angle of repose was measured using the fixed funnel method [24]. An accurately weighed quantity of powdered gum was poured through a funnel. The height of the funnel was adjusted such that its tip just touched the top of the heap of powder below it. The powder was allowed to flow through the funnel freely on to the heap of powder, and the angle of repose was calculated using the following equation:

(2) $$\begin{array}{c} \tan\theta =\frac{h}{r}, \end{array}$$

where *h* is the height of the heap of powder and *r* is the radius of the heap of powder.

#### 2.2.5. Hydration Capacity

The hydration capacity was measured according to the method described by Patel et al. [23]. Powdered NG (1 g) was placed in a 15 mL tube. Then, 10 mL of distilled water was added to the powder, and the mixture was centrifuged for 10 minutes at 1000 rpm. Then the tared centrifuge tube was taken out and inverted to remove the supernatant. The decanted tube was weighed on a digital balance, and the hydration capacity was calculated using the following equation:

(3) $$\begin{array}{c} \text{HC}=\frac{\text{Weight}\;\text{of}\;\text{hydrated}\;\text{sample}}{\text{Weight}\;\text{of}\;\text{dry}\;\text{sample}}. \end{array}$$

#### 2.2.6. Density

The loose bulk density (LBD) and tapped bulk density (TBD) of the NG powder were determined. Powdered gum (5 g) was poured into a calibrated measuring cylinder (10 mL capacity), and the initial volume was noted. Then the cylinder was dropped onto a hard surface from a height of 2.5 cm at 2-second intervals. The tapping was continued until no further change in volume was noted. The LBD and TBD were calculated using the following equation [29]:

(4) $$\begin{array}{c} \text{LBD}=\frac{\text{Weight}\;\text{of}\;\text{the}\;\text{powder}}{\text{Initial}\;\text{volume}\;\text{of}\;\text{the}\;\text{packing}},\\ \text{TBD}=\frac{\text{Weight}\;\text{of}\;\text{the}\;\text{powder}}{\text{Tapped}\;\text{volume}\;\text{of}\;\text{the}\;\text{packing}}. \end{array}$$

#### 2.2.7. Compressibility

The compressibility index (Carr's index) was determined using the following equation:

(5) $$\begin{array}{c} \text{Carr's index}\left(\%\right)=\left[\left(\frac{\text{TBD}\;-\text{LBD}}{\text{TBD}}\right)\right]\times 100. \end{array}$$

#### 2.2.8. Moisture Sorption Capacity

Powdered NG (1 g) was spread uniformly in a petri dish of diameter 9 cm. Then it was maintained at 37 ± 1°C and 100% RH for 2 days in a programmable humidity chamber. The moisture sorption was calculated from the difference between the weights of the sample before and after maintenance in the humidity chamber [24].

### 2.3. Methods of Preparation of SD

A physical mixture (PM) of NG and ATR was prepared by simple blending using a spatula. The PM was passed through a 100# sieve.

#### 2.3.1. Kneading Method

A weighed quantity of a mixture of ATR and NG was placed in a mortar, and the mixture was kneaded thoroughly for 20 minutes with ethanol (1.5 times the amount of mixture). The kneaded mixture was dried in an oven at 60°C until it reached a constant weight, pulverized and screened using a 100# sieve.

#### 2.3.2. Solvent Evaporation Method

ATR was dissolved in ethanol until saturation, and stirring was continued for 30 minutes. NG was suspended in a sufficient quantity of water until a wet mass of polymer was formed. The solution of the drug was poured at into polymer suspension immediately after it was formed. All the solvent was removed by evaporation at 60°C [30].

### 2.4. Characterization of SD

#### 2.4.1. Estimation of Drug Content

Quantities of the physical mixtures (25 mg) produced using the kneading and solvent evaporation methods were dissolved in 25 mL of phosphate buffer solution of pH 6.8. The samples were filtered through a 0.45 *μ*m membrane filter, and the drug content of each was determined spectrophotometrically at 241 nm. Phosphate buffer solution (pH 6.8) was used as the blank.

#### 2.4.2. Solubility Study

The solubility data of ATR, the PM, and the SD prepared using the kneading and solvent evaporation methods in phosphate buffer solution (pH 6.8) were determined. Quantities of the SD equivalent to 50 mg of the drug were added to 100 mL of the buffer in a beaker. The contents of the beaker were stirred for 6 hours using a mechanical stirrer (1000 rpm) at 37 ± 0.5°C. After stirring, the beaker was allowed to stand for 12 hours for equilibration at 37 ± 0.5°C. The resultant solution was filtered through a 0.45 *μ*m membrane filter, and the filtrate was analysed spectrophotometrically at 241 nm.

#### 2.4.3. *In Vitro* Drug Release Study

The dissolution rates of the different SD were determined using 900 mL of phosphate buffer solution (pH 6.8) at 37 ± 0.5°C using a type II USP dissolution test apparatus (EDT-08L-Electrolab, Mumbai, India) run at 75 rpm. Five millilitre aliquots of the dissolution medium were withdrawn at 5, 10, 15, 30, 45, 60, 90, 105, and 120 minutes. The samples were suitably diluted and analysed spectrophotometrically at 241 nm [31]. Different SD was used to determine the dissolution rate of ATR so that the dissolution efficiency (DE) was evaluated using a model-independent approach. DE is defined as the area under the dissolution curve up to a time *t*, expressed as a percentage of the area of the rectangle describing 100% dissolution in the same time. The DE30 and DE120 values were calculated from the dissolution data.

#### 2.4.4. Scanning Electron Microscopy

SEM photomicrographs of ATR and SD prepared using the solvent evaporation method were taken using a scanning electron microscope (JSM 6390, JEOl, Peabody, MA, USA) with a 10 kV accelerating voltage.

#### 2.4.5. Differential Scanning Calorimetry

DSC thermograms of ATR, NG, the PM, and the optimized SD were obtained using a differential scanning calorimeter (DSC 1, Mettler Toledo, Switzerland) with a heating rate of 10°C/minute from 30°C to 300°C in a nitrogen atmosphere.

#### 2.4.6. X-Ray Diffraction Studies

Powder XRD patterns of ATR, NG, and SD were recorded using a diffractometer (PW 1140, Mettler Toledo, Columbus, OH, USA) and Cu-K*α* radiation. The diffractometer was run at a scanning speed of 2°/mm and a chart speed of 2°/2 cm per 2*θ*.

#### 2.4.7. *Ex Vivo* Drug Absorption Study

**Figure 1:**
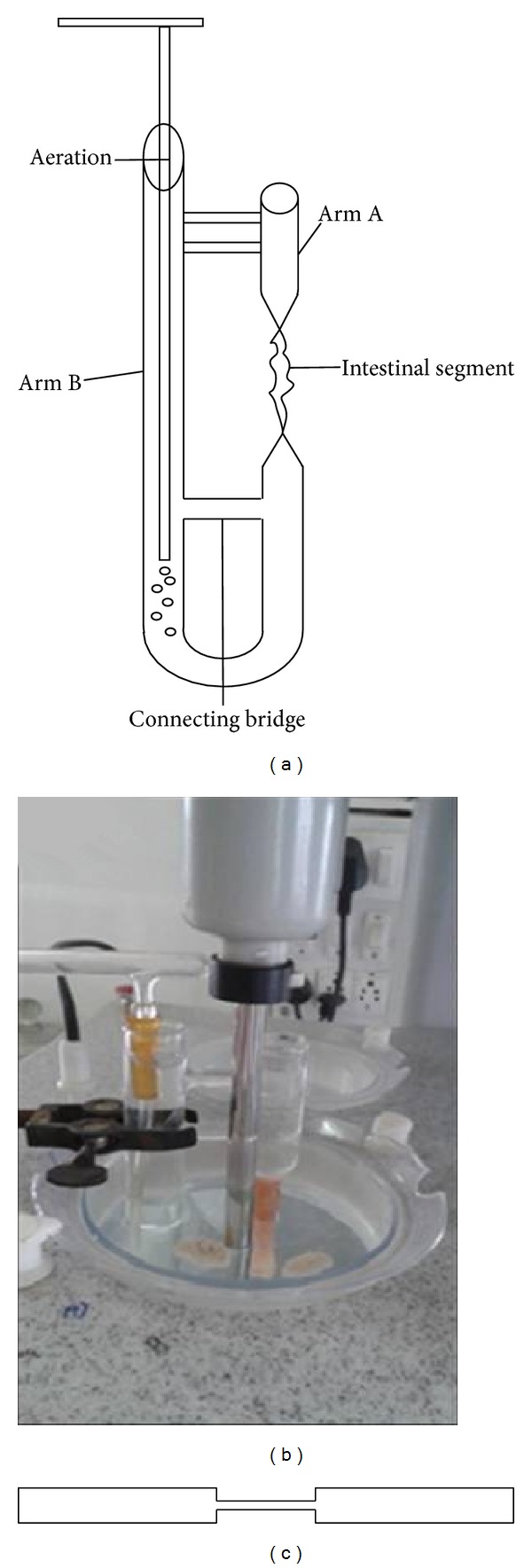
Design of in-house developed continuous dissolution-absorption apparatus (a), photograph of the apparatus with complete setup (b), and glass rod for eversion of chicken ileum (c).

The* ex vivo* absorption profile of each formulation was determined using the modified apparatus (Figure 1) and type II USP dissolution test apparatus (EDT-08L, Electrolab, Mumbai, India). The test was carried out in 1000 mL of phosphate buffer solution (pH 6.8) at 37 ± 0.5°C and a speed of 75 rpm. Five millilitre aliquots of the dissolution medium were withdrawn from arm B at 5, 10, 15, 30, 45, 60, 90, 105, and 120 minutes using a syringe. The samples were analysed spectrophotometrically at 241 nm.

#### 2.4.8. Isolation of Everted Chicken Intestine

Chicken intestine was procured from a slaughter house. The small intestine was taken for the study. The lumen was rinsed with phosphate buffer solution (pH 6.8, Krebs-Ringer solution). A segment of the intestine (6 cm) was taken and transferred to oxygenated phosphate buffer solution (pH 6.8). The intestine was everted using a modified glass rod and tied to the apparatus.

#### 2.4.9. Specifications of Modified Apparatus (Figure 1)

The specifications of modified apparatus were as follows.

1. A connecting bridge (Figure 1(a)) was provided for uniform transfer of absorbed drug from arm A to arm B.
2. Aeration (Figure 1(a)) was carried out to improve the viability of tissues through the oxygen dissolved in the buffer within the apparatus and to mix the dissolved drug particles from the everted intestinal segment uniformly.
3. A modified glass rod (Figure 1(c)) was used to evert the intestinal segment without damage to the inner layer of the intestine.
4. The capacity of the apparatus was 55 mL.

In arm A, the dissolved drug gets absorbed through the everted intestinal segment and mixes with the dissolution medium inside the apparatus. Five millilitre samples were withdrawn at various time intervals and analysed spectrophotometrically at 241 nm.

#### 2.4.10. *In Vivo* Study

An indirect method was used to assess variations in 3-hydroxy-3-methylglutaryl-coenzyme A reductase (NADPH) activity in liver tissue [32]. The HMG CoA and mevalonate concentrations in the tissue homogenate were estimated in terms of the absorbance, and the ratio of the two concentrations was taken as an index of the activity of the enzyme, which catalyses the conversion of HMG CoA to mevalonate. The HMG CoA-to-mevalonate ratio was measured in liver tissues of male albino rats weighing 150–200 g [23].

Rats were divided into three groups (control, standard, and test) of five animals each. All the rats were maintained on a standard diet. For 7 days, the control group was given only a standard diet. The standard group was given a 4 mg/kg dose of ATR with the standard diet, and the test group was given optimized SD equivalent to a 4 mg/kg dose of ATR. After 7 days, the liver tissue was removed as quickly as possible and a 10% homogenate was prepared in saline arsenate solution. The homogenate was deproteinized using an equal volume of dilute perchloric acid. Then it was centrifuged, allowed to stand for 5 minutes, and filtered. To 1 mL of the filtrate, 0.5 mL of a freshly prepared hydroxylamine reagent (alkaline hydroxylamine in the case of HMG-CoA) was added. The filtrate and hydroxylamine reagent were mixed, and 1.5 mL of ferric chloride reagent was added after 5 minutes. The absorbance was read after 10 minutes at 540 nm versus a similarly treated saline arsenate blank. The ratio of HMG-CoA to mevalonate was calculated [33].

#### 2.4.11. Stability Study

The purpose of stability testing is to provide evidence about how the quality of a drug substance or drug product varies with time under the influence of a variety of environmental factors, such as temperature, humidity, and light, and to establish a retest period for the drug substance or a shelf-life for the drug product and recommend storage conditions. Stress testing of the drug substance can help identify the likely degradation products, which can in turn help establish the degradation pathways and the intrinsic stability of the molecule. The nature of the stress testing will depend upon the individual drug substance and the type of drug product involved.

The stability study was carried out to determine the stability of the SD prepared using the kneading and solvent evaporation methods. The SD was sealed in ampoules. These ampoules were maintained at Stability conditions should be 40 ± 2°C and 75 ± 5% RH for 3 months. After this period, the drug content was analysed and* in vitro* drug release studies were carried out.

## 3. Results and Discussion

### 3.1. Polymer Characterization

The results of the NG characterization studies are listed in Table 1. NG has a low viscosity and high water retention capacity. The water retention capacity of a carrier is the amount of water retained in it which indicates the hydrophilic nature of the carrier. Low viscosity of NG makes it suitable candidate for solubility and bioavailability enhancement of poorly water soluble drugs.

### 3.2. Drug Content

The drug content of the SD and the PM is provided in Table 2. The results clearly suggest that the drug content of each formulation is within the theoretical range, indicating that the method used to prepare the formulations is suitable and reproducible in nature.

### 3.3. Solubility Study

The solubility data relating to ATR, the PM, and the SD prepared using the kneading and solvent evaporation methods are listed in Table 3. The drug-to-polymer ratio was optimized by measuring the percent solubility. The percent increases in solubility measured for various drug-to-NG ratios are provided in Table 4. A SD with a drug-to-polymer ratio of 1 : 6 had a significantly enhanced solubility enhancement compared with ATR. The effect of increasing the polymer concentration (drug-to-polymer ratio of 1 : 9) in enhancing the solubility was negligible. Therefore, the optimal ratio of 1 : 6 was selected for further studies.

### 3.4. *In Vitro* Drug Release Study

**Figure 2:**
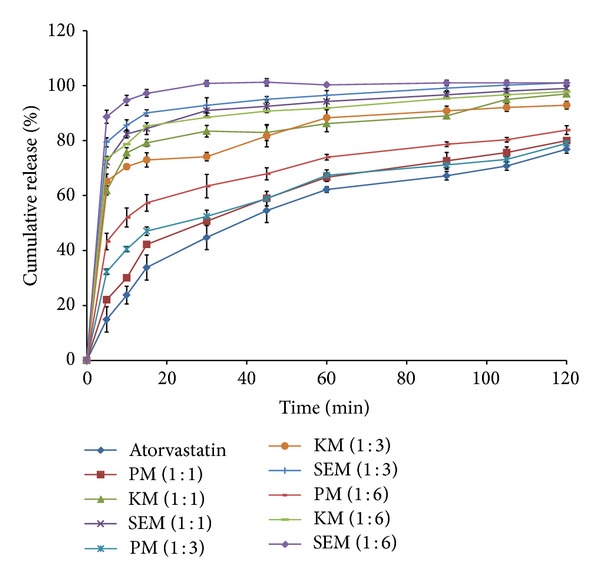
Dissolution profiles of physical mixture (PM), kneading method (KM), solvent evaporation method (SE), and plain atorvastatin.

The* in vitro* drug release profiles of ATR, the PM, and the SD prepared using the kneading and solvent evaporation methods with NG as the hydrophilic carrier are shown in Figure 2. The values of the dissolution efficiency at 30 and 120 minutes are given in Table 5. The SD prepared using the solvent evaporation method displays faster dissolution rates compared with those prepared using the kneading method and the physical mixture (Figure 2). On the basis of results of dissolution efficiency study SD prepared by solvent evaporation method was used for further characterization.

During the process of dissolution, as soon as the drug and polymer particles come in contact with the dissolution fluid, a gel layer of the polymer forms around the drug particles [31]. Thus the diffusion of the dissolved drug through the gelatinous layer is the determining factor in the enhancement of the dissolution rate. From the Stokes-Einstein equation, the diffusion coefficient is inversely proportional to the viscosity. The viscosity of NG was found to be very low (1.763 cps), suggesting that the diffusion of the drug through SD is rapid. During the dissolution process, the drug and polymer particles may get dispersed quickly throughout the dissolution medium, promoting release of the drug.

The dissolution rate of ATR from SD was compared with that of the pure ATR. The dissolution rate of ATR from SD was significantly greater. This may be attributed to the solubilization effect and wetting ability of the hydrophilic carrier. The results indicate that the method of preparation of the SD influences the rate of dissolution of ATR. The dissolution rate is higher with the solvent evaporation method compared with the kneading method and physical blending. The order of increasing dissolution rate was found to be ATR < PM < SD (kneading method) < SD (solvent evaporation method).

### 3.5. Scanning Electron Microscopy (SEM)

**Figure 3:**
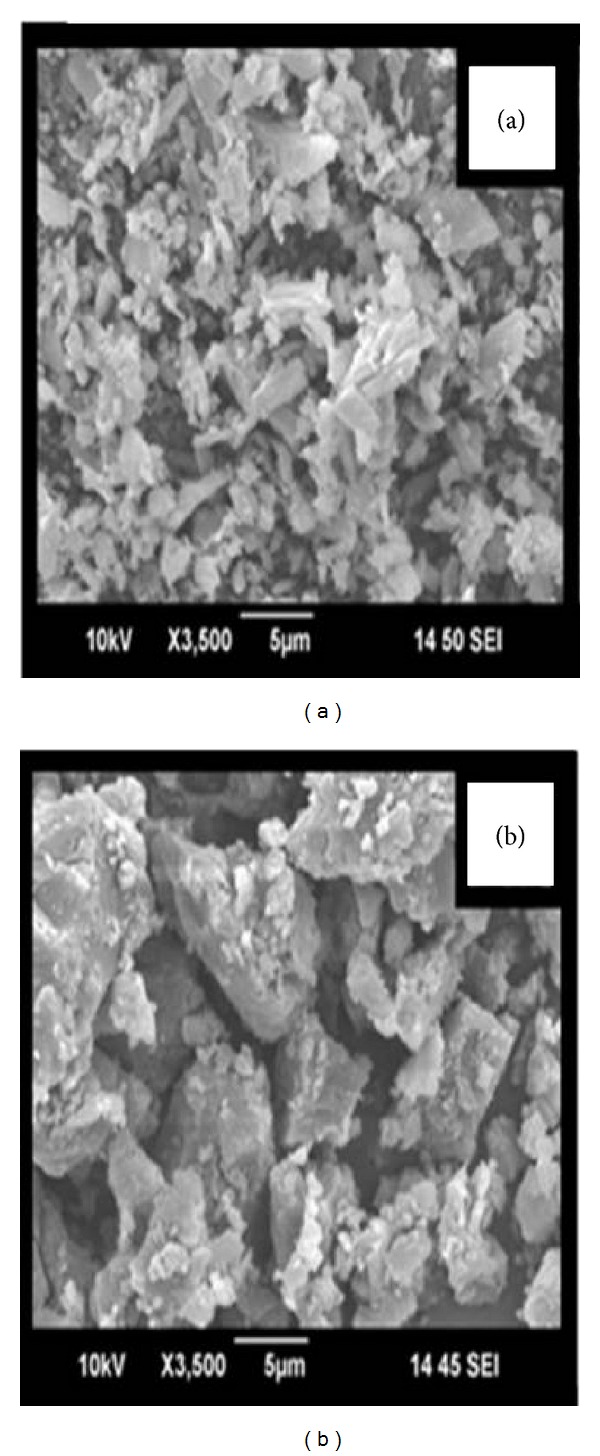
SEM photomicrographs of atorvastatin (a) and its solid dispersion (b).

The scanning electron microscopy photomicrographs of ATR show longer crystals with very specific morphology; in contrast, a decrease in crystallinity was observed with SD prepared using the solvent evaporation method. This was due to the molecular dispersion of ATR in the hydrophilic carrier (Figure 3).

The SEM photomicrographs show that the crystals of ATR are longer and have a plate-like form. In SD prepared by the solvent evaporation method, the crystals form a rough surface in which the drug and polymer are completely fused, forming a uniform single component. The crystalline drug is converted to an amorphous form. This results in a significant increase in the solubility and dissolution rate and thus may improve the bioavailability of ATR. These observations were supported by the results of the DSC and XRD studies.

### 3.6. Differential Scanning Calorimetry

**Figure 4:**
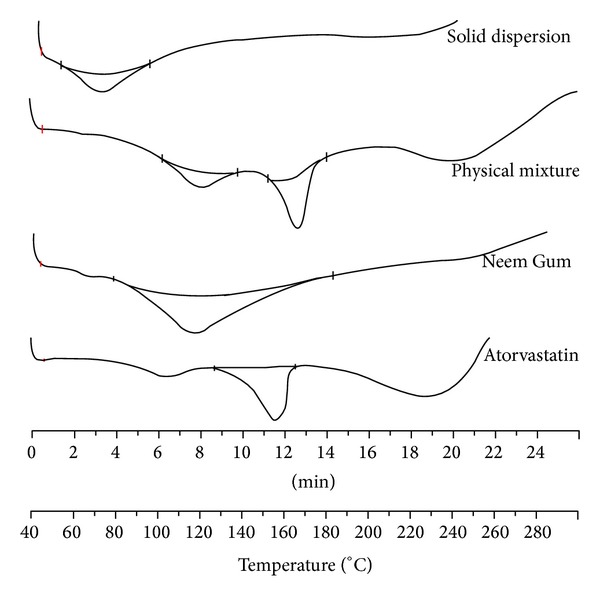
DSC thermograms of atorvastatin, Neem Gum, physical mixture, and solid dispersions.

The DSC thermograms of ATR, NG, PM, and SD (produced using the solvent evaporation method) are shown in Figure 4. The thermograms of ATR exhibit an endothermic peak at 156.42°C, corresponding to its melting point, while NG exhibits a broad endothermic peak owing to its amorphous nature. The endothermic peak of ATR was observed in the thermogram of PM, but it was absent in the thermograms of the SD, suggesting that the crystalline form was converted to the amorphous one.

### 3.7. X-Ray Diffraction Studies

**Figure 5:**
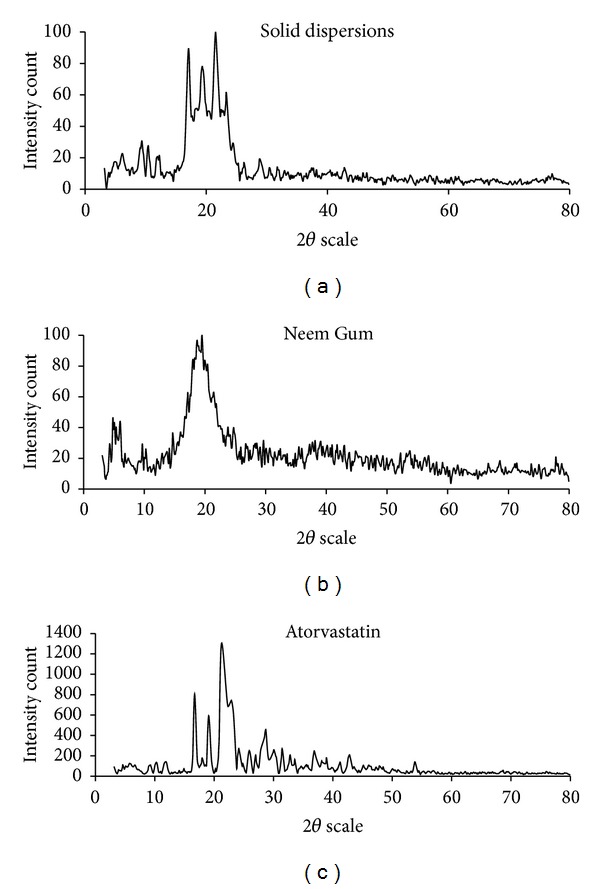
X-ray diffraction studies of atorvastatin, Neem Gum, and solid dispersions.

XRD spectra of pure ATR, NG, and an optimized batch of SD are presented in Figure 5. The X-ray diffractogram of ATR shows sharp and intense peaks at diffraction angles (2*θ*) of 16.721°, 19.091°, 21.248°, 22.286°, and 28.698° suggesting a typical crystalline pattern. Few characteristic crystalline peaks appear in the diffractograms of the SD but at low intensity. This proves that the crystallinity of ATR decreases as most of the drug gets converted to an amorphous form.

### 3.8. *Ex Vivo* Drug Absorption Study Using Modified Apparatus

The permeability of the membrane was evaluated according to the transfer of drug or SD into the intestinal sac as described by Padalkar et al. [30]. Intestinal absorption of a solid state pharmaceutical depends upon two processes that should occur consecutively: (a) dispersion of the pharmaceutical in the gastrointestinal tract and (b) transport of the dispersed pharmaceutical through the blood. When the rate of dispersion of the pharmaceutical is significantly lower than the absorption rate, the dispersion becomes the limiting step in the absorption process. In order to choose techniques or procedures that can increase the solubility, a preliminary study of absorption needs to be carried out. The everted intestinal sac model, when used in the development of a product, allows a comparative evaluation of the behaviour of the pharmaceutical compound during the permeation phase [34].

**Figure 6:**
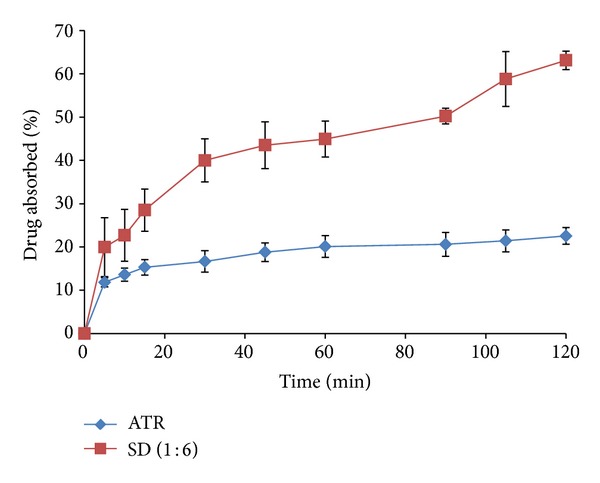
*Ex vivo* absorption data of atorvastatin and solid dispersions.

The* ex vivo* drug absorption study was undertaken with the objective of developing an* ex vivo* continuous dissolution-absorption system using chicken intestinal sac to predict the dissolution-absorption relationships of the SD formulation (solvent evaporation method) and the pure drug (ATR). The results are shown in Figure 6. The percent drug absorption through the intestinal segment of chicken was significantly higher in the case of the SD compared with the pure drug (*P* < 0.05).

### 3.9. *In Vivo* Study

**Figure 7:**
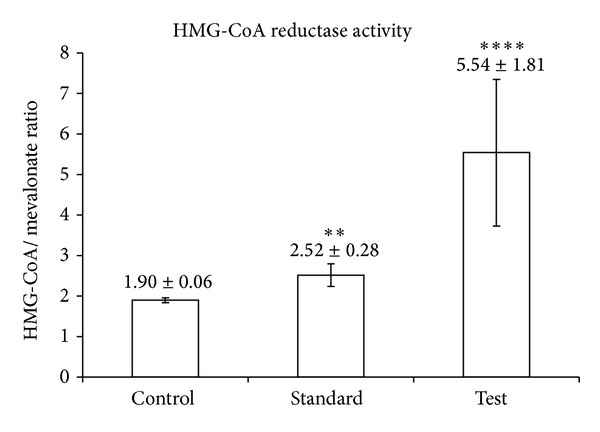
*In vivo* evaluation of solid dispersions.

HMG CoA reductase inhibition activity was measured in terms of absorbance in all the three groups. One-way analysis of variance was used for comparison. All the results are shown as mean ± standard error. The HMG CoA-to-mevalonate ratio of the SD was found to be 5.54 ± 1.81, which was significantly higher than that of plain ATR (2.52 ± 0.28). The *P* value of the SD was found to be less than 0.05, suggesting a significant decrease in enzyme activity compared with plain ATR. This indicates that the performance of the SD is better than that of ATR (Figure 7).

### 3.10. Stability Study

The formulation was found to be stable for 3 months under accelerated testing. There was no significant change in the drug content or dissolution efficiency of the SD prepared using the kneading and solvent evaporation methods (Table 6).

## 4. Conclusion

SD of ATR in a natural hydrophilic carrier, NG, prepared using the solvent evaporation method had significantly improved dissolution rates (*P* < 0.05). The significant wettability, dispersibility, and solubilization effects of NG enhance the solubility and dissolution rate of ATR. The results demonstrate that the optimum ATR-to-NG ratio is 1 : 6. Of the two methods used to prepare solid dispersions, the solvent evaporation method gives a higher dissolution rate. The* in vivo* study indicates that the performance of SD is better than that of ATR as a significant (*P* < 0.05) reduction in the activity of HMG CoA reductase was observed. The* ex vivo* absorption studies conducted on the pure drug (ATR) and SD (solvent evaporation method) suggest that the absorption of ATR from SD is significantly higher compared with pure ATR (*P* < 0.05). In conclusion, NG could be used as a potential natural carrier to enhance the rate of dissolution of ATR, and the modified apparatus developed in-house is a suitable tool for studying* ex vivo* absorption of drugs to predict bioavailability more accurately.

## Figures and Tables

**Table 1:** Characterization of Neem Gum.

Parameters	Neem Gum
Swelling index (%)	159.333 ± 23.43
Water retention capacity (mL)	2.623 ± 0.020
Viscosity (cps)	1.763 ± 0.1908
Angle of repose	31.42 ± 4.088
Hydration capacity	1.082 ± 0.0294
Density	
(1) Bulk density	0.651 ± 0.0721
(2) Tapped density	0.788 ± 0.0246
Carr's index (%)	12.823 ± 0.288
Moisture sorption capacity (%)	5.33 ± 0.577

*n* = 3.

**Table 2:** Estimation of drug content for physical mixture, solid dispersion prepared by kneading, and solvent evaporation method.

Code	Ratios	Atorvastatin (mg)	Polymer (mg)	Drug content %
PM 1	1 : 1	20	20	95.98 ± 0.02
PM 2	1 : 3	20	60	98.74 ± 0.13
PM 3	1 : 6	20	120	100.01 ± 0.04
PM 4	1 : 9	20	180	102.21 ± 0.1
KM 1	1 : 1	20	20	98.85 ± 0.01
KM 2	1 : 3	20	60	98.41 ± 0.17
KM 3	1 : 6	20	120	100.47 ± 0.019
KM 4	1 : 9	20	180	101 ± 0.124
SE 1	1 : 1	20	20	98.35 ± 0.08
SE 2	1 : 3	20	60	99.12 ± 0.4
SE 3	1 : 6	20	120	100.81 ± 0.01
SE 4	1 : 9	20	180	100.32 ± 0.06

*n* = 3; ATR: atorvastatin; PM: physical mixture; KM: kneading method; and SE: solvent evaporation method.

**Table 3:** Solubility studies of atorvastatin (mean ± standard deviation) from physical mixture and solid dispersions in comparison with plain drug.

Product	Solubility (mg/mL)
ATR	0.190 ± 0.04
PM	0.241 ± 0.09
KM	0.746 ± 0.65****
SE	0.843 ± 0.14****

*n* = 3; *P* < 0.05; ATR: atorvastatin; PM: physical mixture; KM: kneading method; and SE: solvent evaporation method.

****refers to significant difference amongst treatment groups.

**Table 4:** Ratio optimization of ATR to NG.

Drug/polymer ratio	% solubility enhancement
1 : 1	63.4
1 : 3	77.6
1 : 6	84.3**
1 : 9	85.8**

**P < 0.05; ATR: atorvastatin; NG: Neem Gum.

**Table 5:** Dissolution efficiency of ATR and various solid dispersions.

Formulation	DE 30 min	DE 120 min
ATR	44.74 ± 4.43	76.86 ± 1.43
PM 1 : 1	50.66 ± 1.52	80 ± 1
PM 1 : 3	52.4 ± 2.13	79 ± 1
PM 1 : 6	63.45 ± 1.95	83.82 ± 2.3
KM 1 : 1	83.43 ± 2.3	97 ± 1.53
KM 1 : 3	74.15 ± 1.53	92.88 ± 1.37
KM 1 : 6	88.47 ± 2.77****	97.84 ± 1
SE 1 : 1	90.96 ± 4.28	99 ± 1.59
SE 1 : 3	92.84 ± 2.45	101 ± 0.277
SE 1 : 6	95.47 ± 1.04****	101 ± 1

*n* = 3, *P* < 0.05.

ATR: atorvastatin; PM: physical mixture; KM: kneading method; and SE: solvent evaporation method.

****refers to significant difference amongst treatment groups.

**Table 6:** Drug content and dissolution efficiency of KM and SE before and after stability.

Formulation	Drug content	DE 30 min	DE 120 min
0 months KM (1 : 6)	100.47 ± 0.12	88.47 ± 2.13	97.84 ± 1.23
After 3 months study KM (1 : 6)	99.84 ± 0.04	86.94 ± 0.42	99 ± 0.014
0 months study SE (1 : 6)	100.81 ± 0.01	100.82 ± 0.13	101 ± 1.23
After 3 months study SE (1 : 6)	100 ± 0.13	100.1 ± 1.42	101 ± 0.014

*n* = 3.

KM: kneading method; SE: solvent evaporation method.
